# Supplementary material for: Gut Microbiota Dysbiosis Is Associated with Elevated Bile Acids in Parkinson’s Disease
Source: Metabolites. 2021 Jan 4;11(1):29. doi: 10.3390/metabo11010029 (PMC7823437; doi:10.3390/metabo11010029)
Supplement: Supplementary file 1 [file metabolites-11-00029-s001.zip › Supplementary File_final/Supplemental Figures_revision.docx]

**Supplemental Figures**

**Gut microbiota dysbiosis is associated with elevated bile acids in Parkinson’s disease**

**Li et al.**

**
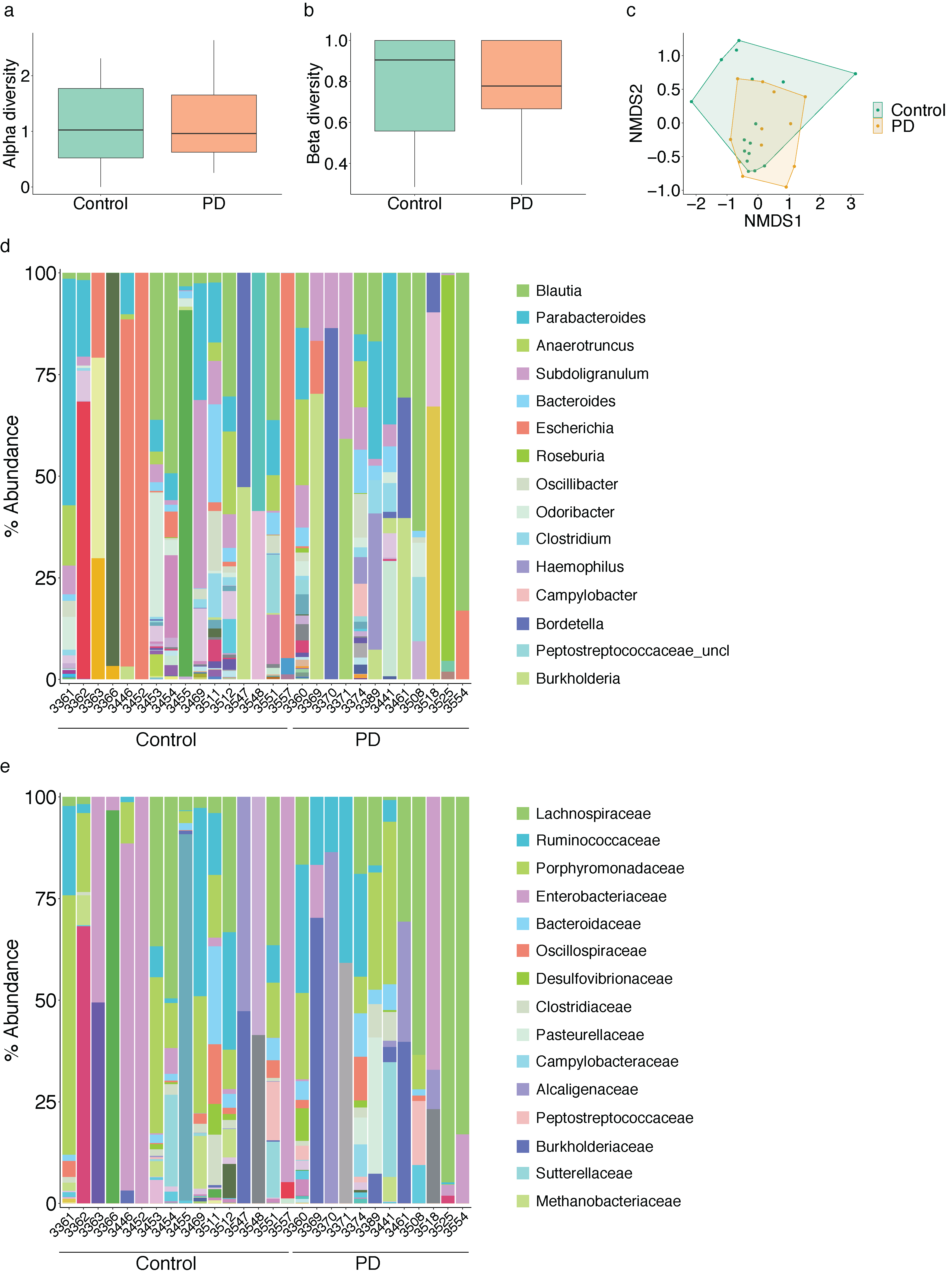
**

**Figure S1.** Microbial diversity in the human appendix. The functional microbiome was determined in metatranscriptomic sequencing data from 12 PD patients and 16 controls. (**a**) Alpha diversity (Shannon index) calculated by vegan package. (**b**) Beta diversity (Whittaker index) calculated by vegan package. (**c**) NMDS plot showing the distribution of samples according to the microbial community. (**d**) Microbiota composition in the human appendix at the genus level. Top 15 most abundant microbiota genera are listed. (**e**) Microbiota composition in the appendix at the family level. Top 15 most abundant microbiota families listed.

**
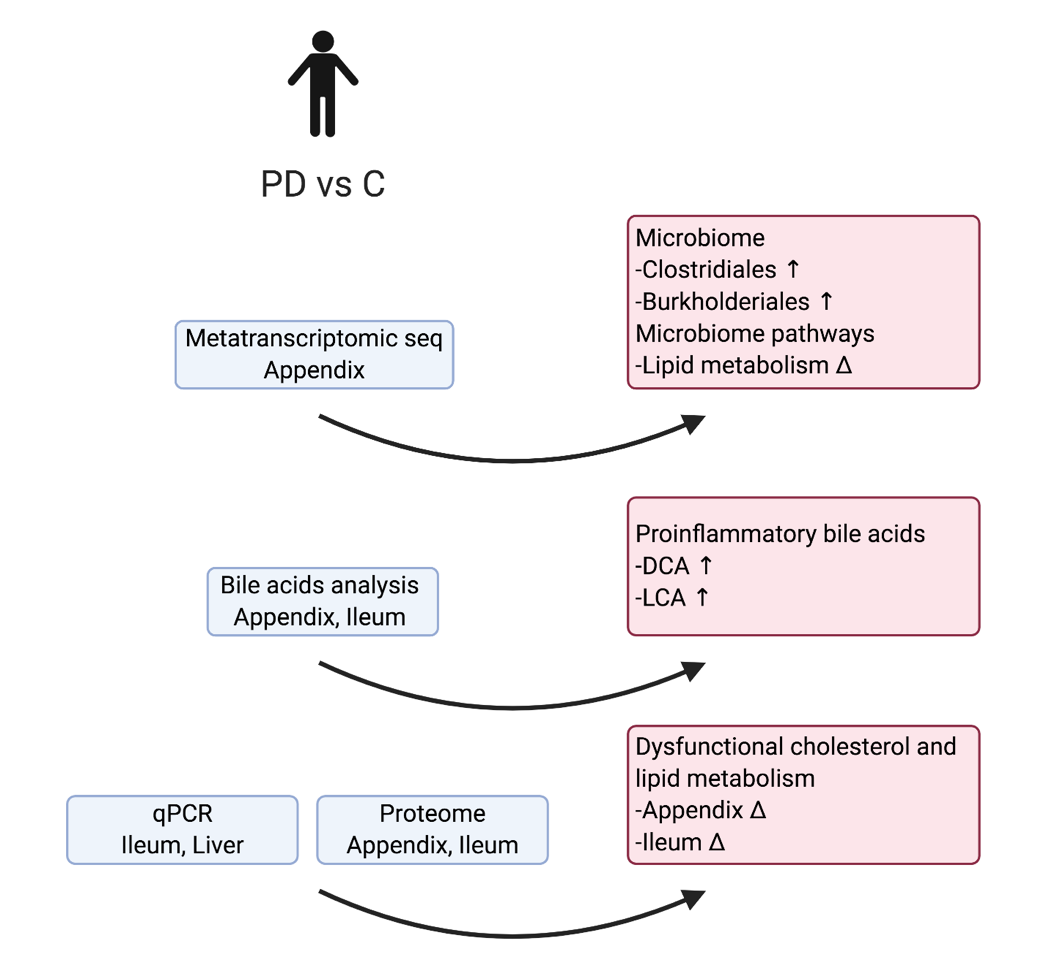
**

**Figure S2: Experimental design and main findings of our study investigating functional changes in the appendix microbiome in PD.** The human appendix is an immunological organ that is also considered to be a storehouse for the gut microbiome. Recently, the appendix has been implicated in the risk of developing PD [1]. To determine whether the PD appendix exhibits functional changes in the microbiome, we performed a metatranscriptomic analysis of the PD and control appendix. We identified microbiome changes in the PD appendix that affect lipid homeostasis and the synthesis of secondary bile acids, which in turn led us to an analysis of bile acids. In the PD appendix, we found an increase in the microbially-derived, cytotoxic bile acids LCA and DCA. Proteomic and transcript analysis also demonstrated a disruption in cholesterol and lipid metabolism in the PD gut.

**
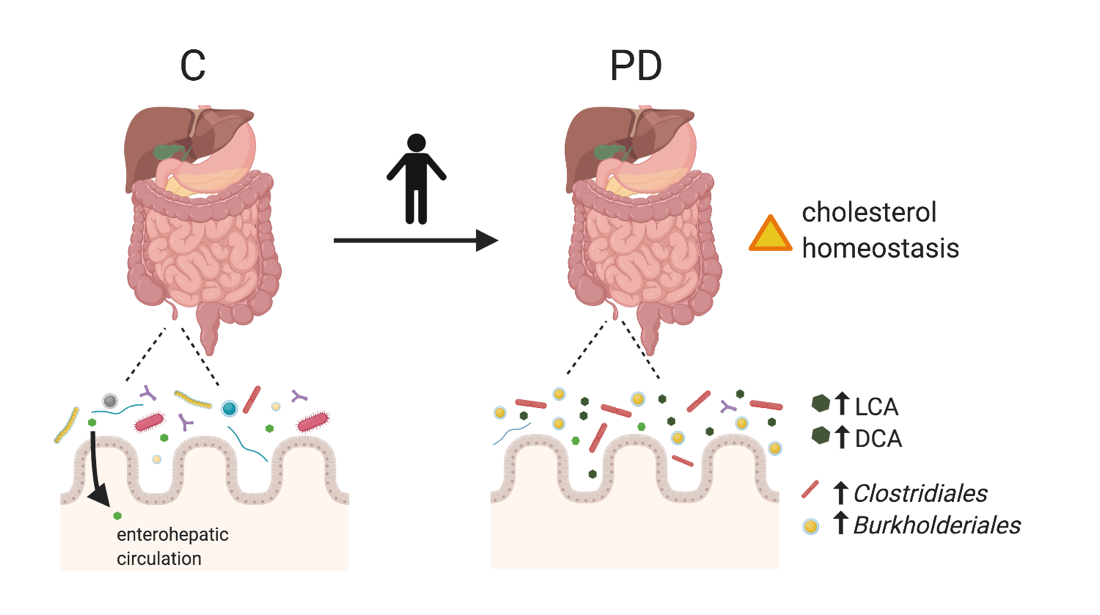
**

**Figure S3: Graphical summary of microbiome and biliary changes in PD.** Microbial dysbiosis and an accompanying increase in microbiome-derived secondary bile acids are prevalent in the PD appendix. Microbiome changes in PD included significant increases in *Clostridiales* and *Burkholderiales,* which are involved in the conversion of primary bile acids to secondary bile acids. Accordingly, elevated secondary bile acids, LCA and DCA, were seen in PD. Transcript and proteomic analyses of the PD gut showed further disruptions in cholesterol metabolism and transport.

**References**

1. Killinger BA, Madaj Z, Sikora JW, et al. The vermiform appendix impacts the risk of developing Parkinson's disease. Science Translational Medicine 2018;10(465).
